# Digital image analysis in pathologist‐selected regions of interest predicts survival more accurately than whole‐slide analysis: a direct comparison study in 153 gastric carcinomas

**DOI:** 10.1002/cjp2.179

**Published:** 2020-09-04

**Authors:** You Jeong Heo, Taebum Lee, Sun‐Ju Byeon, Eun Ji Kim, Hyeong Chan Shin, Binnari Kim, So Young Kang, Sang Yun Ha, Kyoung‐Mee Kim

**Affiliations:** ^1^ The Samsung Advanced Institute for Health Sciences and Technology (SAIHST) Samsung Medical Center, Sungkyunkwan University School of Medicine Seoul Republic of Korea; ^2^ Department of Pathology and Translational Genomics Samsung Medical Center, Sungkyunkwan University School of Medicine Seoul Republic of Korea; ^3^ Department of Pathology Chonnam National University Medical School, Hwasun Hospital Hwasun‐gun Republic of Korea; ^4^ Department of Pathology Hallym University Dongtan Sacred Heart Hospital Hwaseong‐si Republic of Korea; ^5^ Center of Companion Diagnostics Samsung Medical Center Seoul Republic of Korea

**Keywords:** tumor, microenvironment, digital image, automatic, pathology, gastric, cancer

## Abstract

Automatic quantification of biomarkers such as tumor‐infiltrating lymphocytes and PD‐L1 is one of the most studied topics in digital pathology image analysis (DIA). However, direct comparison between the DIA of a whole‐slide image (WSI) and that of regions of interest (ROIs) chosen by pathologists has not been performed. In this study, we aimed to compare the prognostic value of tumor microenvironment markers CD8 and PD‐L1, measured by DIA of WSIs and ROIs. We selected 153 primary gastric cancer tissues and stained them with CD8 and PD‐L1. All IHC slides were scanned at ×200 magnification and ratios of CD8 and PD‐L1 were measured in WSIs and ROIs from the invasive front, within the tumor, and the mucosa. Patients with high CD8 and PD‐L1 ratios showed more favorable outcomes compared to those with low ratios. Pathologist‐aided DIA predicted the survival of patients more accurately than WSI analysis (CD8, *p* = 0.025 versus *p* = 0.068; PD‐L1, *p* = 0.008 versus *p* = 0.2). Although a high density of CD8+ T cells at the invasive front correlated best with patient survival, CD8 ratio in the mucosa could also predict patient outcome. In conclusion, CD8 and PD‐L1 ratios measured by pathologist‐aided DIA predicted survival more accurately than WSI analyses and ROIs at the invasive front correlated best with patient outcome.

## Introduction

The relationship between tumors and their microenvironments has been actively investigated [1], and immune cell infiltration plays an important role in tumor biology [2]. Tumor‐infiltrating lymphocytes (TILs) in cancers correlate well with prognosis [3]. The type, density, and location of immune cells in colorectal cancers have prognostic value superior or comparable to the tumor, node, and metastasis (TNM) stages [4, 5, 6, 7]. Therefore, it is imperative to incorporate immune scores as a prognostic factor and to introduce this parameter as a marker for classifying cancers during routine diagnostic and prognostic assessment of tumors [5]. However, most studies on immune scores were performed and validated in colorectal cancers [6]. Moreover, most studies on TILs in gastric carcinomas (GCs) have been hampered by manual interpretation [8, 9, 10, 11, 12, 13, 14], study with tissue microarrays [9, 11, 15, 16, 17, 18], small numbers of patients [10, 19, 20, 21], or a focus on a specific subtype of immune cells, such as FOXP3+ regulatory T cells [15, 16, 20, 22, 23], CD33+ myeloid cells [12], or CD57+ NK cells [19, 24, 25], rather than CD8+ T cells, the immune cells that most significantly predicted prognosis in recent meta‐analyses of GC data [26, 27]. In GCs, recent meta‐analyses showed that the density of CD8+ T cells [26] and the locations of TILs [27] predicted patient prognosis. However, no study has yet explored TILs in different locations within a single tumor or the effects of location on prognosis.

In addition to predicting prognosis, tumor microenvironment (TME) subtypes based on PD‐L1 status and TILs have emerged as promising biomarkers to predict responses to PD‐1/PD‐L1 pathway blockade [28]. Immunotherapy is a promising approach to GC treatment [29]. In melanomas, selective CD8+ T cell infiltration at the tumor invasive margin and PD‐L1 expression status predict the PD‐1 blockade response [28, 30]. In GCs, TMEs can also be subtyped by PD‐L1 and TILs (represented by CD8+ T cells) [10, 17, 31, 32].

Digital image analysis (DIA) is a rising source of big data for machine learning [33]. Automatic quantification of biomarkers is one of the most studied topics in DIA [34]. Unlike manual interpretation of immunohistochemistry (IHC), which is a subjective, time consuming, and variable process with inherent intraobserver and interobserver variability, DIA offers rapid and uniform interpretation [35]. A recent study of tumor classification and mutation prediction in non‐small cell lung cancer using H&E imaging and deep learning found that DIA offered a significant benefit in the initial diagnosis [36]. Automatic quantification of biomarkers such as tumor‐infiltrating lymphocytes and PD‐L1 is one of the most studied topics in DIA. However, direct comparison between the DIA of a whole‐slide image (WSI) and that of regions of interest (ROIs) chosen by pathologists has not been performed.

In the present study, we selected ROIs from the invasive front, within the tumor, and the mucosa of 153 gastric adenocarcinomas and compared the results with WSI.

## Materials and methods

### Selection of patients

We randomly selected 153 patients who underwent gastrectomy for primary GC at the Samsung Medical Center between 2004 and 2008 and whose data had been used for prior studies (IRB no. 2010‐12‐088) [31, 37]. All patients underwent curative radical gastrectomy with D2 lymph node dissection, with or without adjuvant chemoradiation therapy (INT‐0116 regimen) [38]. Clinical patient data were obtained from electronic medical records during the follow up periods from 2004 to 2012. All patients received curative radical total or subtotal gastrectomy with lymph node dissection, and tumor stage was classified using the American Joint Committee on Cancer (AJCC) Staging Manual, eighth edition. All patients provided informed consent according to Samsung Medical Center institutional guidelines. The patient demographics used for this study are described in supplementary material, Table S1.

### IHC and digital pathology image analysis

IHC staining was performed using Benchmark XT (Ventana, Tucson, AZ, USA) on representative 3 μm sections of formalin‐fixed paraffin‐embedded GC tissues from 153 patients. Each section was deparaffinized in xylene and incubated with rabbit monoclonal anti‐CD8 (clone SP57, Ventana, Tucson, AZ, USA) using a Ventana BenchMark XT autostainer or pharmDx 22C3 PD‐L1 (Agilent Technologies, Dako, CA, USA) using a Dako Autostainer Link 48 as previously described [39].

**Figure 1:**
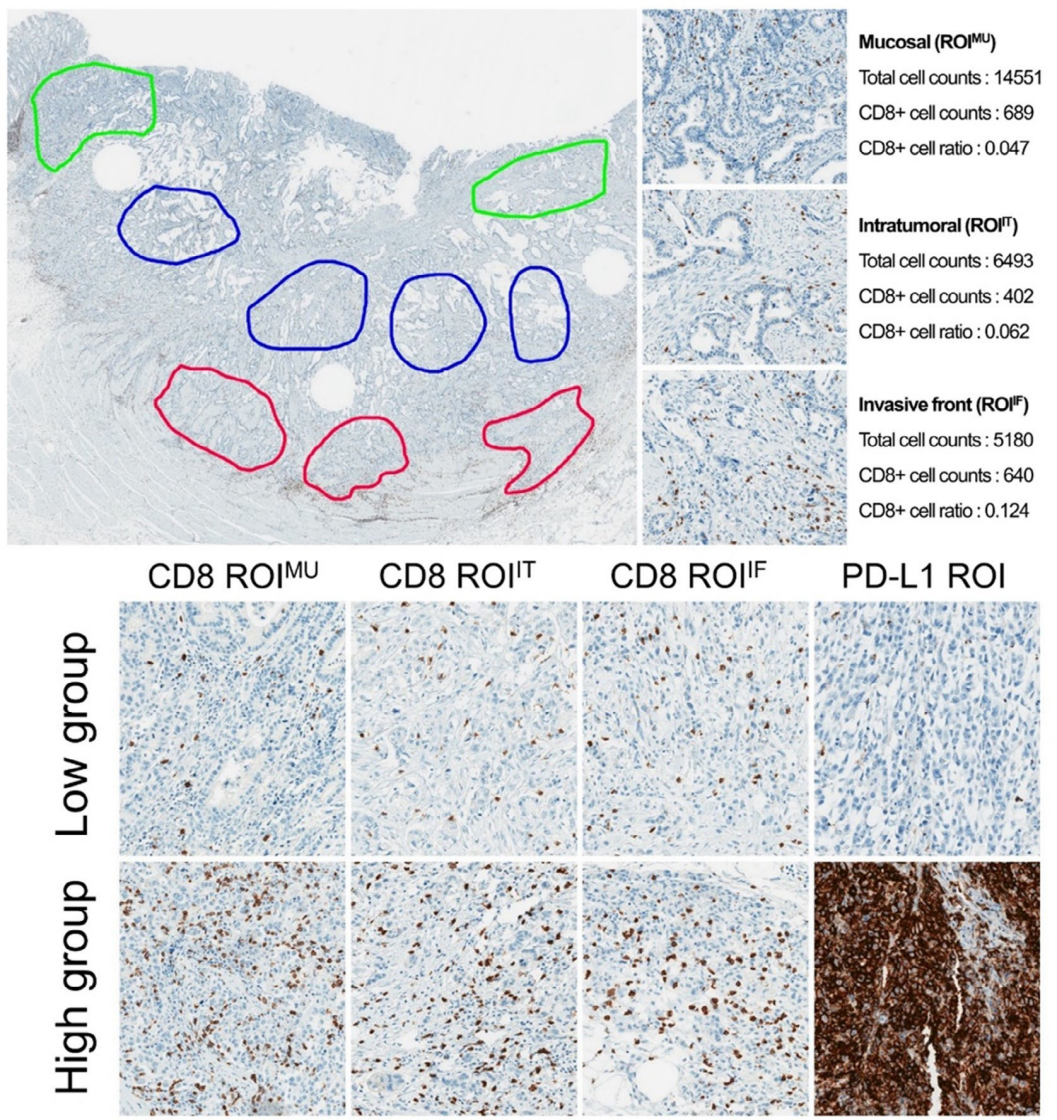
Upper row. Selection of representative ROIs in gastric carcinoma in which to measure CD8 by IHC. Mucosal (green), intratumoral (blue), and invasive front (red) regions were selected. Lower row. Representative photomicrographs of low and high CD8+ and PD‐L1 ratios in the mucosal (ROI^MU^), intratumoral (ROI^IT^), and invasive front (ROI^IF^) regions.

All IHC slides were scanned at ×200 magnification with a ScanScope Aperio AT Turbo slide scanner (Leica Microsystems, Melbourne, Australia). For DIA, we analyzed WSIs and ROIs selected by a pathologist (SJB). For the selection of ROIs, tumor‐rich areas were included, and normal tissue, necrotic tissue, and stroma‐rich areas were excluded. For each slide image, the ROI included mucosal (ROI^MU^), intratumoral (ROI^IT^), and invasive front (ROI^IF^) tumor areas for CD8 (Figure 1, upper row) and a tumor‐rich ROI for PD‐L1. For DIA, the ScanScope Aperio preset nuclear algorithm (Leica) with default parameters was used without modification. For CD8 and CD3, although IHC stained the membranes of T‐cells, we applied nuclear segmentation and quantification algorithms because of their small cell size and sparse cytoplasm as previously described [40]. For PD‐L1, to detect positively stained tumor and immune cells, we applied the ScanScope Aperio cytoplasmic algorithm with default values without modification. Approximately 10 randomly selected fields were chosen from each area and, after careful evaluation of stained slides, absolute numbers of positive cells were counted in each area. The CD8+ and PD‐L1+ cell ratios were calculated by dividing the positive cell counts by the total cell counts (Figure 1, upper row). For comparison, we used the DIA‐whole results (WSI without selection).

### Statistical analysis

R software (version 3.4.4) was used for the statistical analysis. We used the third quartile value of CD8 ratio, CD3 ratio, and PD‐L1 ratio to divide patients into high‐ and low‐CD8 ratio and PD‐L1 ratio groups (Figure 1, lower row). To divide CD8 ratio and CD3 ratio groups in each ROI, the third quartile values of ROI^SUM^ were used as cutoffs. HRs were measured using univariate and multivariate Cox regressions, and an HR forest plot was designed using the forestplot package in R. The Kaplan–Meier method was used for survival curves, and the Wilcoxon rank sum test was used to compare the mean ratio between them. *P* values of <0.05 were considered statistically significant. *K*‐means clustering was performed for dividing patients into two groups using significant factors. Additionally, *k*‐means clustering analysis, an unsupervised clustering algorithm that optimizes the best fit between clusters and their representation using a predefined number of clusters [41], was performed using significant factors in Cox regression analysis. We tested 2, 3, and 4 clusters (*k*). In this study, two clusters (*k* = 2) was selected, which showed the most significant results.

## Results

### Digital pathology image analysis for CD8 and PD‐L1

The CD8 and PD‐L1 ratios in the WSI analyses without annotation (DIA‐whole) and with ROIs selected by a pathologist (DIA‐ROI) were available for all 153 GC cases. The total cell counts ranged from 485 123 to 2 934 741 409 395 (mean, 1 461 677.822) for whole slide images and from 1091 to 216 246 (mean, 71 713.451) for ROIs. The results of the measurements are summarized in Table 1. The median CD8 ratios in the WSIs, ROI^IF^, ROI^IT^, and ROI^MU^ were 0.145 (range, 0.013–0.683), 0.146 (0.011–0.820), 0.128 (0.010–0.828), and 0.077 (0.009–0.698), respectively. The CD8 ratio in WSIs correlated highly with the CD8 ratio in ROIs (mucosal, intratumoral, and invasive front) and correlated most significantly with invasive front (ROI^IF^) regions (see supplementary material, Figure S1). The median values of the PD‐L1 ratio in the whole‐slides and with ROI annotation data were 0.047 (0.008–0.501) and 0.815 (0–0.948), respectively.

**Table 1:** CD8 and PD‐L1 ratios in quantitative image analysis

	Area	Median ratio (range)	Group	*n*	Mean group ratio
CD8	Whole‐slide images	0.145 (0.013–0.683)	High	39	0.506
			Low	114	0.112
	ROI^IF^	0.146 (0.011–0.820)	High	44	0.521
			Low	109	0.118
	ROI^IT^	0.128 (0.010–0.828)	High	44	0.473
			Low	109	0.103
	ROI^MU^	0.077 (0.009–0.698)	High	24	0.441
			Low	129	0.079
PD‐L1	Whole‐slide images	0.047 (0.008–0.501)	High	39	0.231
			Low	114	0.041
	ROI	0.815 (0–0.948)	High	38	0.322
			Low	115	0.010

The MaxStat (maximally selected rank statistics) package of the R programming language (www.r-project.org/) was used to determine the optimal cut‐off points for continuous variables. The third quantile value was chosen for dividing patients into high‐ and low‐CD8 and PD‐L1 ratio groups. After adjusting cut‐off values to the number of cells/mm^2^ area, the results were as follows: 0.06 for PD‐L1 WSI and 0.15 for PD‐L1 ROI; and 619 for CD8 WSI and 1037 for CD8 ROI. As PD‐L1 had fewer positive cells than did CD8 in the analyzed area, the cut off values were very different.

### CD8, PD‐L1, and patient survival

**Figure 2:**
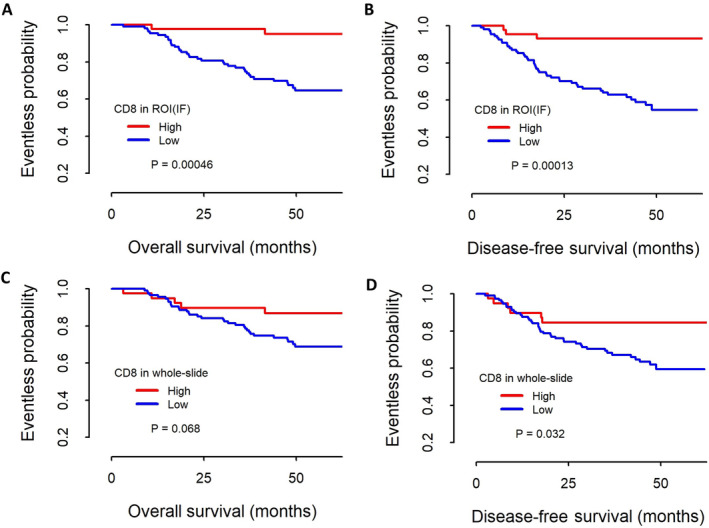
Kaplan–Meier survival curves by CD8 ratio in 153 patients with gastric carcinoma. The high CD8 ratio group showed significantly longer OS and DFS when the invasive front regions were considered (A,B, ROI^IF^, *p* < 0.001 for both OS and DFS), but the difference was less significant in the WSIs (C,D, *p* = 0.068 for OS and 0.032 for DFS).

The CD8^HIGH^ group showed significantly longer overall survival (OS) and disease‐free survival (DFS) than the CD8^LOW^ group using DIA‐ROI^SUM^ (*p* = 0.025 in OS and 0.0044 in DFS) and all DIA‐ROI. This difference was most significant in the DIA‐ROI^IF^ data (*p* = 0.00046 for OS and 0.00013 for DFS) (Figure 2A,B). Analysis of DIA‐whole also showed favorable outcomes, but the differences were not statistically significant (*p* = 0.068 for OS and 0.032 for DFS) (Figure 2C,D).

**Figure 3:**
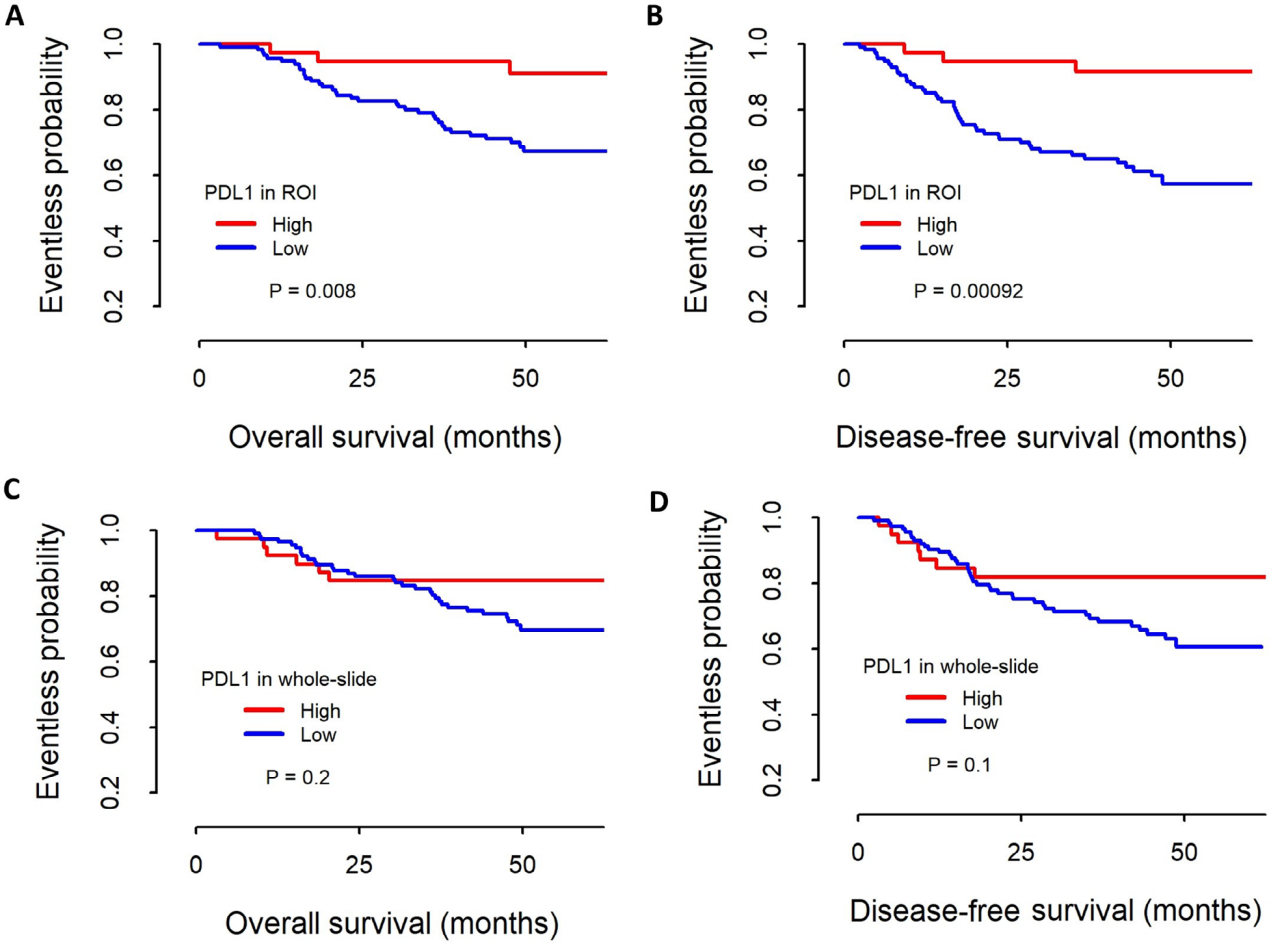
Kaplan–Meier survival curves for the PD‐L1 ratio in 153 patients with gastric carcinoma. Patients with a high PD‐L1 ratio in selected tumor‐rich areas (A,B) and WSIs (C,D) showed significantly longer OS and DFS. Although the difference was significantly different in the tumor‐rich areas selected by a pathologist (*p* = 0.008 for OS and <0.001 for DFS), this significant difference was lost in the WSIs (*p* = 0.2 for OS and 0.1 for DFS).

The PD‐L1^HIGH^ group showed significantly longer OS and DFS using both DIA‐ROI and DIA‐whole data (Figure 3). However, the predictive power was more significant for results from DIA‐ROI (*p* = 0.008 for OS and 0.00092 for DFS) than for results from DIA‐whole (*p* = 0.2 for OS and 0.1 for DFS).

To test classification using both CD8 ROIs (ROI^IF^, ROI^IT^, and ROI^MU^) and PD‐L1 ROI, we clustered two patient groups using the *k*‐means clustering method. [41] The cluster 1 group had significantly longer OS and DFS than the cluster 2 group (*p* = 0.0073 for OS and 0.0019 for DFS) (see supplementary material, Figure S2).

### Association of CD3+ and CD8+ T cells with clinicopathologic features of gastric adenocarcinomas

To better characterize the association of the host immune responses against gastric adenocarcinoma, we performed analyses on CD3+ and CD8+ T cells with clinicopathological features. The CD8 and CD3 ratios in ROIs, selected by a pathologist (DIA‐ROI), were available in 196 GC cases from the Asian Cancer Research Group (ACRG) study cohort [37] and the Cox proportional hazard modeling results with clinicopathologic variables are summarized in supplementary material, Table S2. Patients were divided into high‐ and low‐CD3 and CD8 ratio groups using the third quantile values.

The CD8^HIGH^ group showed significantly longer OS and DFS than the CD8^LOW^ group using DIA‐ROI^SUM^ (*p* = 0.004 for OS and 0.0067 for DFS) and all DIA‐ROI. This difference was most significant in the DIA‐ROI^IF^ data (*p* = 0.0036 for OS and 0.004 for DFS). However, there were no differences between the CD3^HIGH^ and CD3^LOW^ groups.

As with CD8 and PD‐L1, we clustered 2 patient groups using the *k*‐means clustering method to test classification using CD3 ROIs (ROI^IF^, ROI^IT^, and ROI^MU^) and CD8 ROIs (ROI^IF^, ROI^IT^, and ROI^MU^). The cluster 2 group had significantly longer OS and DFS than the cluster 1 group (*p* = 0.0053 for OS and 0.0222 for DFS) (see supplementary material, Figure S3).

### Cox proportional hazard models to predict OS and DFS

The CD8 ratio and PD‐L1 in DIA‐ROI, EBV positive and TNM stages were all independent prognostic factors predicting patient outcomes in the univariate analysis and HR forest plot (Table 2). For the CD8 ratio, CD8^LOW^ in DIA‐ROI^IF^ was a more significant predictor of shorter OS (*p* = 0.003; HR = 8.369) and DFS (*p* = 0.001, HR = 7.086) than ROI^SUM^, ROI^MU^, or ROI^IT^ (see supplementary material, Figure S4). PD‐L1^LOW^ in DIA‐ROI was also a significant predictor of shorter OS (*p* = 0.015; HR = 4.316) and DFS (*p* = 0.003; HR = 5.739) (Table 2). For multivariate analysis, EBV, TNM stages, CD8 ROIs, and PD‐L1 ROIs were selected, which were significant predictors in univariate analysis. TNM stages, especially stage IV, and CD8 ROI^IF^ were independent prognostic factors in multivariate analysis (Table 3). CD8^LOW^ in DIA‐ROI^IF^ was a significant predictor of shorter OS (*p* = 0.023; HR = 10.308) and DFS (*p* = 0.027, HR = 6.762).

**Table 2:** Univariate analysis in Cox proportional hazard modeling

		Overall survival		Disease‐free survival	
		HR (95% CI)	*P* value	HR (95% CI)	*P* value
Age (years)	≥60 versus <60	0.976 (0.513–1.859)	0.942	1.123 (0.632–1.997)	0.692
Sex	Female versus male	1.169 (0.598–2.286)	0.647	0.981 (0.531–1.813)	0.951
Location	Cardia versus antrum	1.054 (0.290–3.832)	0.936	0.739 (0.215–2.536)	0.630
	Body versus antrum	1.302 (0.610–2.783)	0.495	0.987 (0.521–1.868)	0.967
	Whole and multiple versus antrum	2.494 (0.852–7.302)	0.095	1.554 (0.569–4.243)	0.390
Lauren	Diffuse versus intestinal	1.260 (0.406–3.922)	0.687	1.080 (0.398–2.944)	0.876
	Mixed versus intestinal	1.380 (0.685–2.797)	0.365	1.070 (0.576–1.976)	0.837
EBV	Positive versus negative	0.174 (0.042–0.722)	**0.016**	0.204 (0.063–0.658)	**0.008**
MSI	MSI‐H versus. MSS	0.872 (0.268–2.836)	0.820	0.890 (0.319–2.485)	0.825
AJCC stages	Stage II versus Stage I	2.880 (0.612–13.57)	0.181	4.220 (0.935–19.05)	0.061
	Stage III versus. Stage I	5.909 (1.380–25.31)	**0.017**	7.646 (1.813–32.24)	**0.006**
	Stage IV versus Stage I	19.26 (4.071–91.13)	**<0.001**	19.35 (4.093–91.49)	**<0.001**
CD8	Whole, low versus high	2.339(0.913–5.993)	0.077	2.476 (1.051–5.833)	0.038
	ROI^SUM^, low versus high	3.076 (1.091–8.67)	**0.034**	3.969 (1.425–11.06)	**0.008**
	ROI^IF^, low versus high	8.369 (2.015–34.77)	**0.003**	7.086 (2.199–22.83)	**0.001**
	ROI^IT^, low versus high	2.822 (1.102–7.231)	**0.031**	2.996 (1.272–7.059)	**0.012**
	ROI^MU^, low versus high	7.966 (1.092–58.09)	**0.041**	10.04 (1.384–72.8)	**0.023**
PD‐L1	Whole, low versus high	1.765 (0.738–4.222)	0.202	1.94 (0.869–4.331)	0.106
	ROI, low versus high	4.316 (1.327–14.04)	**0.015**	5.739 (1.781–18.49)	**0.003**

Significant *P* values are shown in bold.

**Table 3:** Multivariate analysis in Cox proportional hazard modeling

		Overall survival		Disease‐free survival	
		HR (95% CI)	*P* value	HR (95% CI)	*P* value
EBV	Positive versus negative	0.740 (0.125–4.382)	0.740	0.791 (0.192–3.264)	0.746
AJCC stage	Stage II versus Stage I	2.291 (0.466–11.244)	0.307	3.101 (0.673–14.298)	0.147
	Stage III versus Stage I	3.986 (0.881–18.044)	0.073	4.544 (1.043–19.787)	0.044
	Stage IV versus Stage I	11.043 (2.166–56.302)	**0.004**	11.056 (2.248–54.366)	**0.003**
CD8	ROI^SUM^, low versus high	0.243 (0.033–1.786)	0.165	0.337 (0.058–1.958)	0.226
	ROI^IF^, low vesus high	10.308 (1.379–77.042)	**0.023**	6.762 (1.242–36.812)	**0.027**
	ROI^IT^, low versus high	1.080 (0.219–5.318)	0.925	0.822 (0.240–2.813)	0.754
	ROI^MU^, low versus high	1.578 (0.111–22.474)	0.737	2.407 (0.212–27.286)	0.478
PD‐L1	ROI, low versus high	1.651 (0.467–5.834)	0.437	2.459 (0.716–8.425)	0.152

Significant *P* values are shown in bold.

## Discussion

### Technical aspects of digital immune cell analysis, DIA, and deep learning

DIA is a new source of big data for machine learning in medicine [33]. However, there are obstacles to adopting DIA in clinical practice [31]. To support routine clinical application of automatic quantification of TME and TILs, we directly compared the results of DIA‐whole and DIA‐ROI in the same cohort and found that using DIA in areas selected by a pathologist improved its predictive power dramatically. In a previous study, we found that human interpretation was superior to DIA in classifying TME subtypes and predicting patient prognosis [31]. Given that manual interpretation is costly and inherently more subjective than DIA [34], we used pathologist‐selected tumor‐rich areas within the scanned images for the DIA and found that DIA‐ROI showed much stronger power to predict DFS and OS than DIA‐whole.

### CD8 and PD‐L1 as biomarkers in gastric cancers

The relationship between a tumor and its TME is critical for tumor growth and metastasis and has therefore been actively investigated [1]. The clinical significance of immune infiltration in cancer was suggested [4, 5] and validated by an international consortium study in a large cohort of colorectal cancer patients [6]. In GC, several previous studies have analyzed the levels of immune cell infiltration and examined their potential clinical relevance [10, 26]. However, those studies were limited by the use of manual interpretation instead of DIA [8, 9, 10, 11, 12, 13, 14], tissue microarray study [9, 11, 15, 16, 17, 18], small sample size [10, 19, 20, 21], and lack of a validation set [42]. Recently, Jiang *et al*. [42] developed immune‐based classification (Immunoscore), a prediction model for GC patients using CD3 ^IF^, CD3 ^IT^, CD8 ^IF^, CD45RO ^IT^, and CD66b ^IF^ data from 879 consecutive patients. They showed clinical significance of the immune cells, but their prediction model requires manual interpretation by two pathologists with at least 89% agreement, five representative areas, and five IHC procedures. The abundance of immune and other cells in the TME has been estimated by computational methods using gene expression data [43, 44, 45, 46, 47]. However, gene expression data are difficult to apply in the clinic, and *in silico* immune context is critical for successful immunotherapy. Therefore, we tried to classify the TME in GCs to better understand tumor–immune interactions and facilitate patient selection for future immunotherapy using CD8+ T cells and PD‐L1 that are currently measured using manual interpretation by pathologists [31] and computational measurements [40]. We used digital measurements of CD8+ T cells at the invasive fronts and PD‐L1 in the tumor‐rich areas, and we used FDA‐approved PharmDx kits for PD‐L1 IHC, the most significant biomarker for immunotherapies targeting the PD‐1/PD‐L1 pathway, in a large GC cohort with full tissue sections of gastric adenocarcinoma to investigate clinicopathological characteristics and their effects on prognosis. Similar studies have been reported in several cancers including triple‐negative breast cancers [48, 49]. Although these studies measured TILs using company‐based software, not an instrument‐based algorithm like ours, they found substantial variability in CD8+ TILs between individual patients and across the nine types of human cancer although their effects on prognosis are not described [3].

### The role of CD8 particularly in gastric cancers

The prognostic role of CD8+ TILs has been actively investigated in many tumor types. Piras *et al*. [50] evaluated the density of CD8+ lymphocytes (low, 0–20 cells/HPF; moderate, 20–100; high, >100) at the base of the tumor mass in 47 patients with Stage I and II primary cutaneous melanoma, and found that patients with high CD8+ TIL density showed longer OS than that of others (*p* = 0.01). In colorectal cancer, Nosho *et al*. [51] utilized 768 cases and quantified the density of intratumoral CD8+ cells using TMA and DIA and found that patients with high CD8+ TILs were significantly associated with longer cancer‐specific survival (*p* = 0.007). The density of CD8+ TILs in non‐small cell lung cancer (NSCLC) has also been studied as a promising prognostic tool. A study with 797 NSCLC patients using four different cohorts from Norway and Denmark [52] scored the percentages of CD8+ T cells compared to the total numbers of nucleated cells in the tumor stroma, and concluded that tumor stromal CD8+ TILs were an independent prognostic factor for DFS and OS in multivariate analysis (*p* < 0.001). A recent study addressing the role of CD8 in predicting response to nivolumab showed significantly longer progression‐free survival (*p* = 0.0002) while PD‐L1 expression was not associated with survival benefits [53]. Moreover, Immunoscore has been regarded as a good predictor of responses to various therapeutic modalities [54].

Recent studies on GC have shown that the high density of CD8+ T cells at the tumor invasive margin correlates highly with patient survival [3, 42]. Given the favorable prognosis of GC patients [26], infiltration of immune cells, especially CD8+ T cells, into tumor tissues clearly causes physical destruction of tumor cells, reduces tumor burden, and improves clinical prognosis through direct physical contact between tumor cells and the infiltrated immune cells [55]. In this context, the locations of the infiltrating immune cells, in addition to the TIL subtypes, matter. Although high levels of CD8 T‐cells in tumors have been linked to positive clinical outcomes more commonly than the levels of any other cell type, and in a number of different tumors, the precise localization of CD8 T‐cells within the tumor also alters their prognostic significance. In the present study, we also found that the CD8 ratio in ROI^IF^ was the most significant factor for predicting longer OS and DFS. In addition, we are the first to identify that ROI^MU^ could also be a good immunomarker to predict patient outcomes.

### Digital technology and the necessity of human pathological interaction

With this direct comparison study on gastric adenocarcinomas, the prediction of prognosis was superior in DIA‐ROI than in DIA‐whole. This result is attributable to enriched tumor cells, so the diluting effects of normal tissue, necrotic tissue, and stromal cells are diminished, and thus this result provides more useful information about the disease state. Moreover, we found that confining the image analysis region from WSI to ROI creates an accurate and computationally viable method for tissue image analysis. Based on these observations, we could prove that pathologists with the knowledge of biology, histology, pathology, pathophysiology, biomarker expression, and comparative anatomy play an important role in the era of DIA and artificial intelligence (AI).

The present study has several limitations. Although we validated the results of CD8 analysis in different cohorts from the same institute, we could not validate our results in patients with gastric cancer at other centers. Moreover, during the measurement of WSI, we did not filter the morphological or technical artefacts (bubbles or folded tissue), or parts of the normal tissue such as normal gastric mucosa or lymphoid follicles, which interfere with the results. Although DIA provided accurate and reproducible quantitative data, different analytic methods, and selection of variable ROIs, would affect the cutoff values and alter the clinical significance.

In conclusion, DIA‐ROI was superior to DIA‐whole and although ROI^IF^ was the most significant factor to predict prognosis, ROI^MU^ also predicts patient outcomes.

## Author contributions statement

YJH, TL, SYK, SYH and K‐MK designed the study. S‐JB selected ROIs for digital analysis. EJK scanned slides. HCS and BK selected patients and collected clinical information. YJH performed digital pathology analysis and statistical analysis. YJH, TL and K‐MK wrote the manuscript. All the authors interpreted and discussed the results and provided critical comments to the manuscript.

## Supporting information


**Figure S1.** Correlation between whole slide images (WSIs) and regions of interest (ROIs)
**Figure S2.** Kaplan–Meier survival curves for the PD‐L1 and CD8 ratios
**Figure S3.** Kaplan–Meier survival curves for the CD3 and CD8 ratios from the ACRG cohort
**Figure S4.** Forest plot of the Cox proportional hazard model
**Table S1.** Patient demographics used for this study
**Table S2.** Association of CD3+ and CD8+ T cells with clinicopathologic features in the ACRG cohort
