# Supplementary material for: Digital image analysis in pathologist‐selected regions of interest predicts survival more accurately than whole‐slide analysis: a direct comparison study in 153 gastric carcinomas
Source: J Pathol Clin Res. 2020 Sep 4;7(1):42–51. doi: 10.1002/cjp2.179 (PMC7737754; doi:10.1002/cjp2.179)
Supplement: Supplementary file 1 — Figure S1. Correlation between whole slide images (WSIs) and regions of interest (ROIs) Figure S2. Kaplan–Meier survival curves for the PD‐L1 and CD8 ratios Figure S3. Kaplan–Meier survival curves for the CD3 and CD8 ratios from the ACRG cohort Figure S4. Forest plot of the Cox proportional hazard model Table S1. Patient demographics used for this study Table S2. Association of CD3+ and CD8+ T cells with clinicopathologic features in the ACRG cohort [file CJP2-7-42-s001.docx]

**Digital image analysis in pathologist-selected regions of interest predicts survival more accurately than whole-slide analysis: a direct comparison study in 153 gastric carcinomas**

Heo YJ *et al*, *J Pathol Clin Res*, DOI 10.1002/cjp2.179

**Supplementary Material**

**Supplementary Figures**


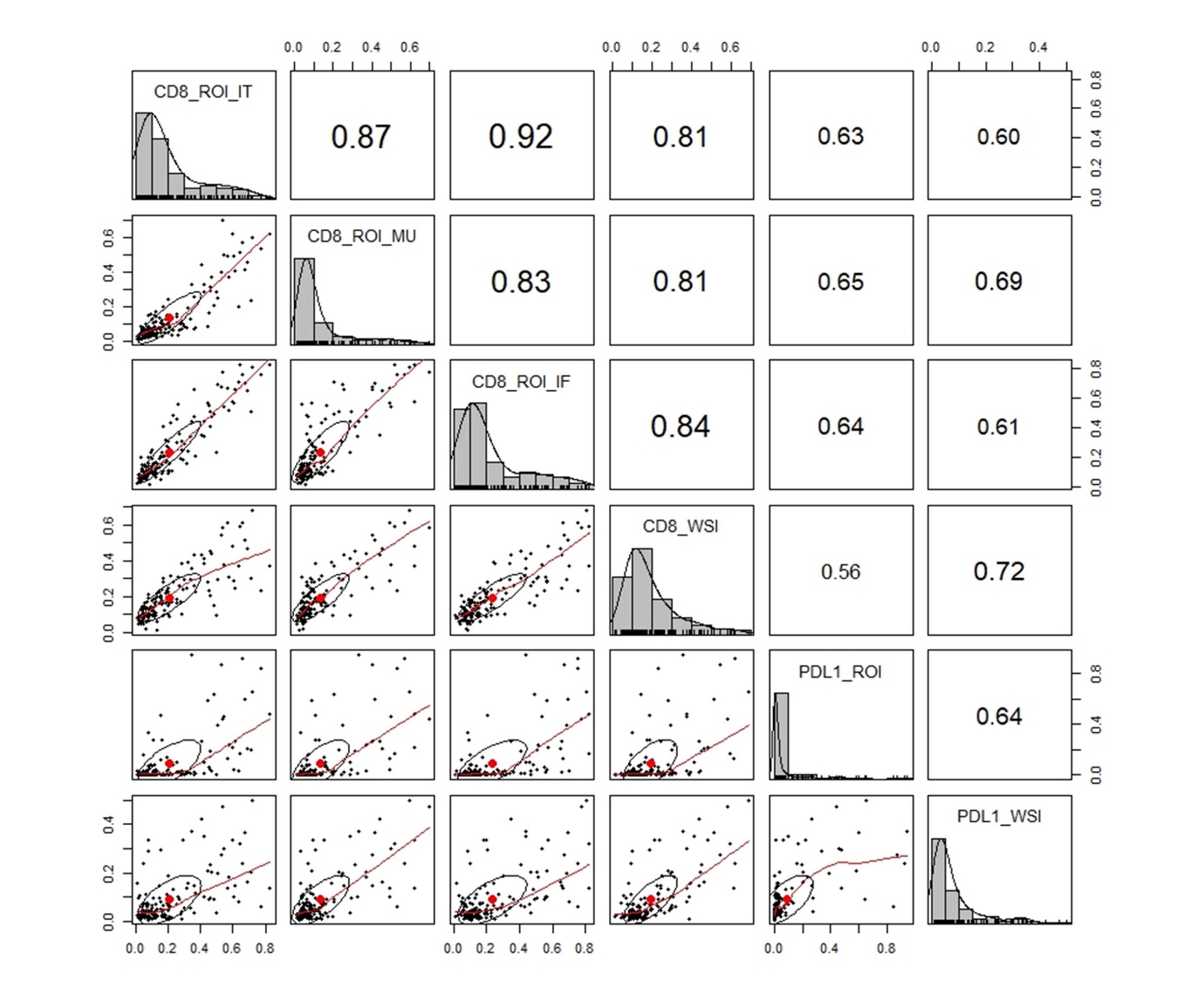


**Figure S1.** Correlation between whole slide images (WSIs) and regions of interest (ROIs). CD8 ratio in WSIs correlated highly with CD8 ratio in ROIs (mucosal, intratumoral and invasive front), especially in invasive front regions.

**
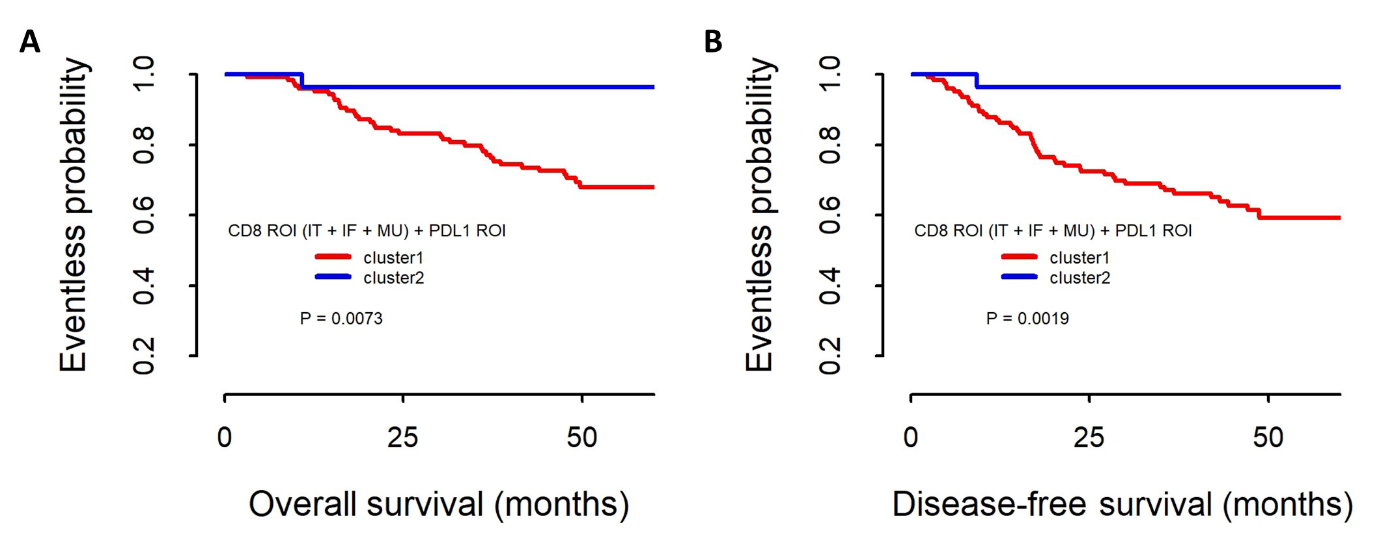
**

**Figure S2**. Kaplan-Meier survival curves for the PD-L1 and CD8 ratios in 153 patients with gastric carcinoma. Patients with cluster 1 groups showed significantly longer OS and DFS than in cluster 2 groups (p=0.0019 in OS and 0.1 in DFS).


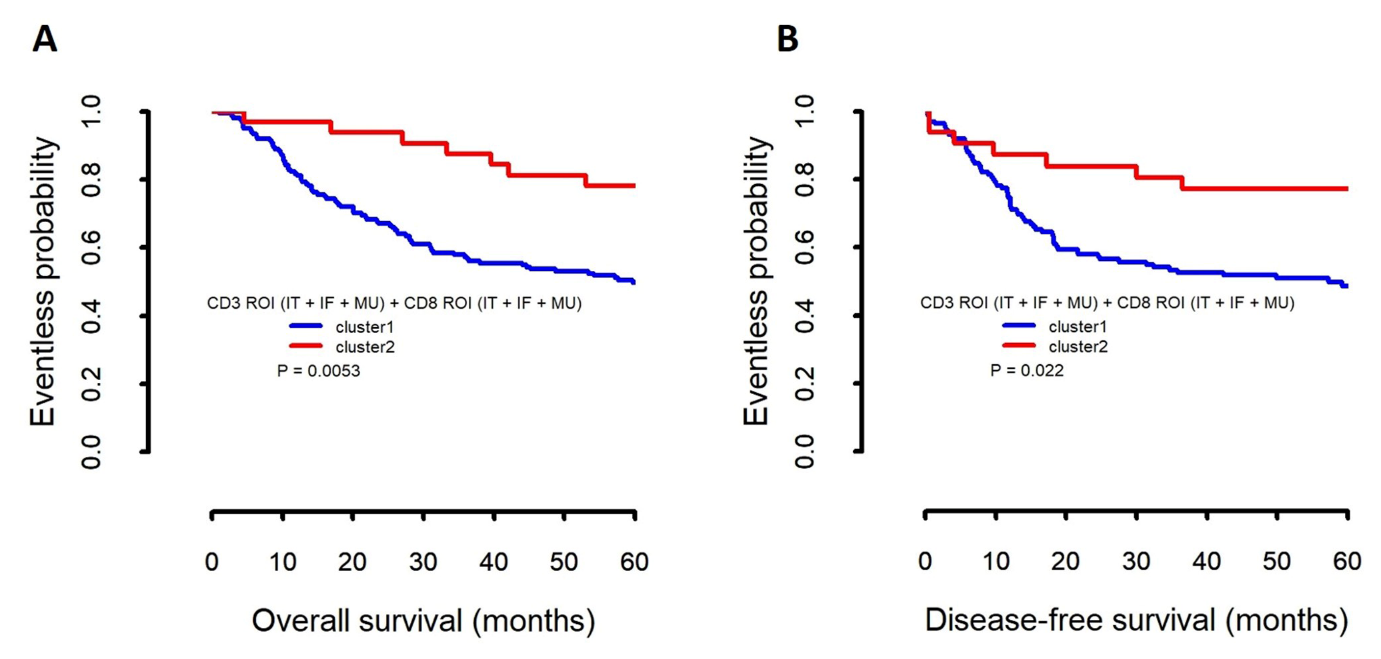


**Figure S3.** Kaplan-Meier survival curves for the CD3 and CD8 ratios in 196 patients with gastric carcinoma from the ACRG cohort. Patients in the cluster 2 groups showed significantly longer OS and DFS than those in the cluster 1 groups (p=0.0053 for OS and 0.022 for DFS).

­­­­­


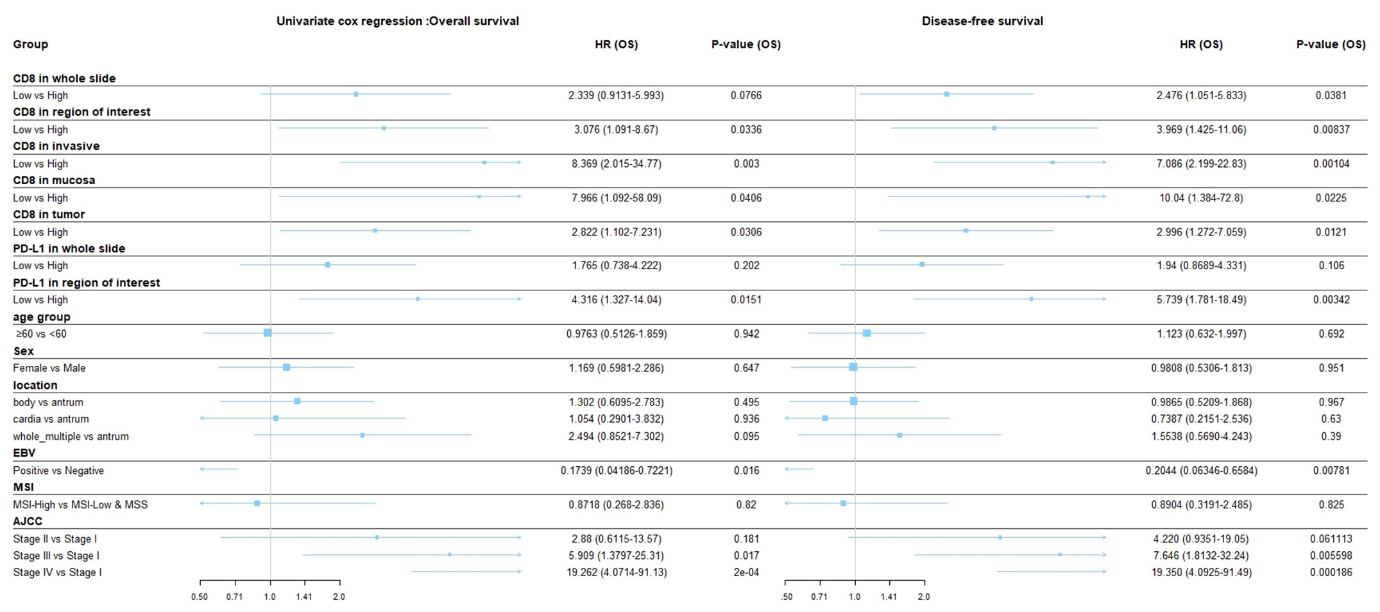


**Figure S4.** Forest plot of the Cox proportional hazard model. The group with a low CD8 ratio in the invasive front had the highest hazard ratio (HR) in OS (p=0.003; HR=8.369) and DFS (p=0.001; HR=7.086). The PD-L1 ratio in the region of interest (ROI) also correlated significantly with OS (p=0.015; HR=4.316) and DFS (p=0.003; HR=5.739).

**Supplementary Tables**

**Table S1**. Patient demographics used for this study.

| Patient characteristics | Numbers (%) |
| --- | --- |
| Age |  |
| ≥60 | 66 (43) |
| <60 | 87 (57) |
| Sex |  |
| Male | 107 (70) |
| Female | 46 (30) |
| Lauren histology |  |
| Intestinal | 55 (36) |
| Diffuse | 16 (11) |
| Mixed | 74 (48) |
| Indeterminate | 8 (5) |
| EBV status |  |
| Negative | 118 (77) |
| Positive | 35 (23) |
| MSI status |  |
| MSS | 139 (91) |
| MSI-H | 14 (9) |
| HER2 status |  |
| Positive | 12 (8) |
| Negative | 141 (92) |
| TNM/AJCC stages |  |
| I | 30 (20) |
| II | 47 (31) |
| III | 65 (42) |
| IV | 11 (7) |
| Recurrence |  |
| No | 106 (69) |
| Yes | 47 (31) |
| Died of disease |  |
| No | 115 (75) |
| Yes | 38 (25) |

**Table S2.** Association of CD3+ and CD8+ T cells with clinicopathologic features of gastric adenocarcinomas in the ACRG cohort.

|  | | Overall survival | | Disease-free survival | |
| --- | --- | --- | --- | --- | --- |
|  |  | HR (95% CI) | *P*-value | HR (95% CI) | *P*-value |
| Age, years | ≥60 vs. <60 | 1.381 (0.901-2.116) | 0.138 | 1.100 (0.706-1.714) | 0.674 |
| Sex | Female vs. Male | 1.395 (0.915-2.125) | 0.122 | 1.219 (0.771-1.928) | 0.397 |
| Location | Cardia vs. Antrum  Body vs. Antrum  Whole & multiple vs. Antrum | 1.044 (0.662-1.646)  1.477 (0.809-2.696)  0.897 (0.279-2.890) | 0.853  0.205  0.856 | 1.272 (0.787-2.057)  1.493 (0.760-2.934)  0.629 (0.151-2.613) | 0.326  0.245  0.523 |
| Lauren | Diffuse vs. Intestinal  Mixed vs. Intestinal | 1.711 (1.130-2.593)  2.064 (0.739-5.769) | **0.011**  0.167 | 1.680 (1.075-2.626)  2.307 (0.708-7.521) | **0.023**  0.166 |
| EBV | Positive vs. Negative | 0.853 (0.373-1.952) | 0.707 | 0.837 (0.339-2.071) | 0.701 |
| MSI | MSI-H vs. MSS | 0.520 (0.222-1.215) | 0.131 | 0.377 (0.137-1.038) | 0.059 |
| AJCC stages | Stage II vs. Stage I  Stage III vs. Stage I  Stage IV vs. Stage I | 2.259 (0.513-9.950)  6.475 (1.559-26.890)  15.127 (3.649-62.710) | 0.281  **0.010**  **<0.001** | 1.856 (0.411-8.380)  6.111 (1.461-25.560)  14.963 (3.591-62.350) | 0.422  **0.013**  **<0.001** |
| CD3 | ROI^SUM^, Low vs. High  ROI^IF^, Low vs. High  ROI^IT^, Low vs. High  ROI^MU^, Low vs. High | 1.379 (0.841-2.261)  1.202 (0.7772-1.871)  0.998 (0.642-1.553)  1.282 (0.748-2.197) | 0.202  0.414  0.994  0.366 | 1.288 (0.763-2.175)  1.189 (0.738-1.915)  1.049 (0.647-1.701)  1.331 (0.759-2.33) | 0.344  0.478  0.846  0.319 |
| CD8 | ROI^SUM^, Low vs. High  ROI^IF^, Low vs. High  ROI^IT^, Low vs. High  ROI^MU^, Low vs. High | 2.206 (1.269-3.835)  2.082 (1.258-3.447)  1.864 (1.137-3.057)  2.470 (1.242-4.914) | **0.005**  **0.004**  **0.014**  **0.010** | 2.284 (1.236-4.221)  2.233 (1.274-3.915)  2.303 (1.294-4.098)  2.469 (1.188-5.130) | **0.008**  **0.005**  **0.005**  **0.015** |

Abbreviations: ROI, region of interest; IF, invasive front; IT, intratumoral; MU, mucosal. Significant *P*-values are shown in bold.
